# Creating a data resource: what will it take to build a medical information commons?

**DOI:** 10.1186/s13073-017-0476-3

**Published:** 2017-09-22

**Authors:** Patricia A. Deverka, Mary A. Majumder, Angela G. Villanueva, Margaret Anderson, Annette C. Bakker, Jessica Bardill, Eric Boerwinkle, Tania Bubela, Barbara J. Evans, Nanibaa’ A. Garrison, Richard A. Gibbs, Robert Gentleman, David Glazer, Melissa M. Goldstein, Hank Greely, Crane Harris, Bartha M. Knoppers, Barbara A. Koenig, Isaac S. Kohane, Salvatore La Rosa, John Mattison, Christopher J. O’Donnell, Arti K. Rai, Heidi L. Rehm, Laura L. Rodriguez, Robert Shelton, Tania Simoncelli, Sharon F. Terry, Michael S. Watson, John Wilbanks, Robert Cook-Deegan, Amy L. McGuire

**Affiliations:** 1 American Institutes for Research, Health Care Group, 100 Europa Drive, Suite 315, Chapel Hill, NC 27517 USA; 2 Center for Medical Ethics and Health Policy, Baylor College of Medicine, One Baylor Plaza, Houston, TX 77030 USA; 3 Deloitte, 1919 N Lynn, Arlington, VA 22209 USA; 4 Children’s Tumor Foundation, 120 Wall Street, 16th Floor, New York, NY 10005 USA; 5 Department of English, Concordia University, 1455 Boulevard de Maisonneuve O, Montreal, QC H3G 1M8 Canada; 6 Human Genetics Center, School of Public Health, The University of Texas Health Science Center at Houston and Human Genome Sequencing Center, Baylor College of Medicine, One Baylor Plaza, Houston, TX 77030 USA; 7 Faculty of Health Sciences, Simon Fraser University, 8888 University Drive, Burnaby, BC V5A 1S6 Canada; 8 Law Center and Department of Electrical and Computer Engineering, University of Houston, 4604 Calhoun Road, Houston, TX 77004 USA; 9 Treuman Katz Center for Pediatric Bioethics, Seattle Children’s Research Institutes, 1900 Ninth Avenue, Room 677, Seattle, WA 98101 USA; 10 Human Genome Sequencing Center, Baylor College of Medicine, One Baylor Plaza, Houston, TX 77030 USA; 11 23andMe, 899 West Evelyn Avenue, Mountain View, CA 94041 USA; 12 Verily Life Sciences LLC, 269 E. Grand Avenue, South San Francisco, CA 94080 USA; 13 Milken Institute School of Public Health, The Georgetown Washington University, 950 New Hampshire Avenue, NW, Second Floor, Washington, DC, 20052 USA; 14 Center for Law and the Biosciences, Stanford University, Neukom N361, Stanford, CA 94305 USA; 15 Illumina, Inc., 5200 Research Pl, San Diego, CA 92122 USA; 16 Centre of Genomics and Policy, McGill University, Montreal, 740 Avenue Drive Penfield, Suite 5200, Montreal, Quebec H3A 0G1 Canada; 17 Institute for Health and Aging, and Department of Anthropology, History, and Social Medicine, University of California San Francisco, San Francisco, CA 94143 USA; 18 Department of Biomedical Informatics, Harvard Medical School, 10 Shattuck Street, Boston, MA 02115 USA; 19 Kaiser Permanente, University of California San Diego and Singularity University, 4965 Maynard Street, San Diego, CA 92122 USA; 20 Center for Population Genomics, Boston Veterans Administration Healthcare, 73 Mount Wayte Avenue, Framingham, MA 01702 USA; 21 Duke University School of Law; Duke Innovation and Entrepreneurship Initiative, 210 Science Drive, Box 90360, Durham, NC 27708 USA; 22 Harvard Medical School, Brigham and Women’s Hospital and The Broad Institute of MIT and Harvard, 65 Landsdowne Street, Cambridge, MA 02139 USA; 23 Division of Policy, Communications, and Education, National Human Genome Research Institute 31 Center Drive, Room 4B09, Bethesda, MD 20892 USA; 24 Private Access, Inc, 19800 MacArthur Boulevard, Suite 300, Irvine, CA 92612 USA; 25 Broad Institute of Harvard and MIT, 415 Main Street, Cambridge, MA 02142 USA; 26 Genetic Alliance, 4301 Connecticut Avenue, NW, Suite 404, Washington, DC, 20008 USA; 27 American College of Medical Genetics and Genomics, 7101 Wisconsin Avenue, Suite 1101, Bethesda, MD 20814 USA; 28 Sage Bionetworks, 1100 Fairview Avenue N., Mailstop M1-C108, Seattle, WA 98109 USA; 29 School for the Future of Innovation in Society and Consortium for Science, Policy and Outcomes, Arizona State University, and Senior Fellow, FasterCures, a Center of the Milken Institute, 1834 Connecticut Avenue, NW, Washington, DC, 20009 USA

## Abstract

National and international public–private partnerships, consortia, and government initiatives are underway to collect and share genomic, personal, and healthcare data on a massive scale. Ideally, these efforts will contribute to the creation of a medical information commons (MIC), a comprehensive data resource that is widely available for both research and clinical uses. Stakeholder participation is essential in clarifying goals, deepening understanding of areas of complexity, and addressing long-standing policy concerns such as privacy and security and data ownership. This article describes eight core principles proposed by a diverse group of expert stakeholders to guide the formation of a successful, sustainable MIC. These principles promote formation of an ethically sound, inclusive, participant-centric MIC and provide a framework for advancing the policy response to data-sharing opportunities and challenges.

## Background

In 2011, a National Academies report advocated for the creation of an “Information Commons,” an individual-centric, multilayered, widely accessible informational resource to support integration and use of data from biomedical research and clinical care for precision medicine [1]. This report built on the spirit of open science embodied in the publicly funded Human Genome Project and its Bermuda Principles, which were modeled on data-sharing practices in nematode biology and called for daily sharing of DNA sequence data well before publication [2]. Since then, data initiatives facilitating clinical and genomic data sharing have proliferated across multiple sectors and internationally. The term “commons” in this context evokes the groundbreaking work of Nobel laureate Elinor Ostrom, who developed a theoretical framework to address governance challenges in building and sustaining shared natural resources [3]. She and others then extended the concept to “knowledge commons,” applying to resources that are not necessarily depleted by use [4]. This framework has informed sophisticated commentary on the challenges of data sharing in the healthcare context [5].

One lesson from Ostrom’s work is that a successful, sustainable commons requires stakeholder participation. With health data, stakeholder engagement is required to address long-standing policy concerns about data access, privacy and security protections, data ownership, and issues of data quality, interoperability, and network sustainability. Without a nuanced understanding of these complexities and a policy framework to address them, it will be difficult to obtain stakeholder buy-in and to realize the promise of advancing precision medicine and promoting a learning healthcare system.

In an effort to enact Ostrom’s lessons and to begin to address these critical issues, we have joined together as representatives of a range of stakeholders, including healthcare systems, clinical laboratories, technology companies, academia, government, nongovernmental organizations, and patient and community advocacy groups. Drawing on our first-hand experiences, we have created an initial list of eight core principles (1–8) for building a successful, sustainable medical information commons (MIC), defined as “a networked environment in which diverse health, medical, and genomic data on large populations become broadly available for research use and clinical applications”.

## Core principles

### 1. The MIC should be a healthy “ecosystem” of data initiatives connected through a standard approach to policy, interoperability, and collaborative work

The MIC does not refer to a particular network architecture, but rather to an ecosystem that encompasses multiple actors and a comprehensive, high-level, stakeholder-informed framework to guide decision making about data control and access. Some of the initiatives contributing to the MIC may have their own policies and governance structures for sharing data. Thus, the larger MIC may encompass multiple smaller commons, as defined by Elinor Ostrom. Rather than attempting to create uniformity, the preferred approach is to develop core principles (including the eight set forth here) and policies that ensure the needs and concerns of key stakeholders are considered and addressed across different models. Building the MIC ecosystem will require not only the creation of appropriate stakeholder-specific incentives for sharing data, but also new approaches in how large-scale data-sharing policies are developed and implemented by academia, industry, and government. The Global Alliance for Genomics and Health and the Patient-Centered Outcomes Research Network are current examples of experiments in multistakeholder policy development.

### 2. The MIC must bring together diverse sources of data from individuals with different states of health

An optimal MIC would include diverse sources of data from a broad range of individuals. Data (phenome and demographics) would be obtained from electronic health records, genomes, and personal health and environmental sources, including wearables, and would be voluntarily shared by individuals and continuously updated throughout their lives. Including data from a broad range of individuals is essential to enable analysis of disease risk, identification of factors contributing to disease resistance/resilience, and testing of pharmacogenomic targets. Aggregation could be accomplished through multiple strategies, including: linking data from current or planned large-scale population-based initiatives (like the Precision Medicine Initiative’s All of Us Research Program) and research efforts by health systems (such as Kaiser-Permanente and Partners HealthCare); linking disease-specific repositories; directing data from existing cohorts into a centralized data repository; and compiling searchable metadata into a directory or index that enables authorized inquiries to be directed to the location where responsive data are held.

Furthermore, targeting recruitment of individuals in various states of health would provide a rich data source. Researchers and clinicians would have access to information to understand the full spectrum of disease etiologies and define the natural history of disease, accelerate the development of more accurate diagnoses and treatments, and reduce the difficulty, time, and expense of identifying and recruiting relevant cohorts for clinical studies. However, interoperability and adopting data-sharing policies that maximize the utility of the data across heterogeneous sources are major challenges for the MIC. The variation between the many electronic health record formats created by different companies, customized for different health systems, and tailored for different purposes from medical value to ease of billing, magnify these challenges.

### 3. A participant-centric model is critical for the sustainability of the MIC

The existing opaque system of exchanging data built solely around data holders and users must transition to a system that involves participants. Involvement may require that the system is built with the participant at the center, or at least require a system that more meaningfully empowers the individuals whose data are being shared to make decisions about access and use [6, 7]. Empowerment requires not just giving participants choices but helping them understand their choices in a very technical area. Policy development should focus on options that simplify the system as much as possible, including the possibility of inserting trusted intermediaries to navigate the complex politics involved in data exchange [8]. Tools such as Blockchain, FHIR, Sync for Science and Sync for Genes, Private Access, and Blue Button can be refined and/or created to facilitate the ability of individuals to contribute and control data in the MIC. Use of these tools promotes autonomous and informed decision making without requiring that individuals own their data in a legal sense or exercise exclusive unstructured control over access to their data (see principle 8).

If data-sharing initiatives subscribing to the participant-centric model are to thrive within the MIC, strategies supporting an individual’s choice to contribute data directly to the MIC must be developed. These strategies should be simple, convenient, dynamic, community-based, and grounded in an ethical foundation that builds and sustains trust by being trustworthy. Existing US law generally gives individuals the right to access their healthcare data and requires covered entities to share it with others (including in electronic form), but more work is needed to help individuals exercise this right and to implement authorized data access and transfer [6, 7]. As the MIC evolves, community-driven and community-based efforts will likely increase in number, and it will be important that these data repositories are connected to and interoperable with traditional institutionally governed initiatives.

The participant-centric model must also integrate the rights, legal obligations, and interests of other stakeholders, including researchers, clinicians, and institutions. To promote cooperation among these various stakeholders, incentives to share data (or not) must be analyzed and, where appropriate, augmented (for example, rewarding researchers in the areas of promotion, publication credit, and tenure for sharing data).

### 4. It is important to reach out to and engage under-represented populations and to investigate the feasibility and acceptability of a public health approach

Relying on individuals to voluntarily contribute their data through existing healthcare delivery and communication channels will not be enough to engage a truly diverse group of participants. The situation is further complicated by the historical mistrust of research based on past injustices experienced by underserved groups, such as Native Americans and African Americans, which may result in under-representation of minority populations in the MIC. Under-representation will weaken the scientific generalizability and clinical utility of the resulting resources by failing to address some of the most genetically diverse communities, thus missing important scientific insights and further exacerbating health disparities.

A public health approach featuring automatic enrollment coupled with opt-out mechanisms and robust protections against data misuse might help ensure a representative cohort and address concerns about selection bias. Such an approach, however, raises concerns about unjustified and unwanted infringement on individual liberties. Systematic outreach and alternative engagement methods and strategies to invite communities to join the commons are needed to achieve equitable representation in the MIC. Some communities have possible channels for engagement through trusted intermediaries and community representatives, such as tribal governments and the Genetic Resource Center of the National Congress of American Indians, or via “community engagement studios” [9, 10].

### 5. Building trust is an iterative process and requires investment of efforts beyond informed consent

Building trust is essential for a successful MIC. Fostering and maintaining trust involves active engagement, takes time, and requires keen tuning to people’s needs, as trust can easily be damaged and, once lost, it is difficult to restore. Potential ways to build and sustain trust with participants include meaningful and authentic engagement through trusted intermediaries (including advocacy groups and foundations) or giving participants a meaningful voice in governance and/or decisions regarding their data.

Simply relying on traditional requirements for informed consent is insufficient, especially when obtaining consent is treated as a transaction rather than an iterative, ongoing process of communication. Participant choices can change over time as circumstances change. Such evolution supports the adoption of dynamic, process-oriented engagement and consent. Other trust-building factors include transparent communication of data distribution and uses, clear data-sharing and distribution rules, and meaningful sanctions for misuse. Demonstration of the financial viability of data repositories would minimize participants’ data security concerns related to disruption of funding or potential bankruptcy.

### 6. Regulatory policies that rely on a sharp distinction between the “kingdom of research” and the “kingdom of clinical care” must be reconsidered

The current regulatory system clearly distinguishes research data from clinical data. Researchers and clinicians are governed by different legal rules and ethical norms when collecting, storing, and using health data, depending on whether those data were collected as part of a research study or as part of clinical care. This distinction is far less meaningful for participants. As long as they are provided protections against informational harms, participants are mainly concerned that the data promote progress toward disease prevention and treatment and improvements for themselves or others in the future. The MIC ideally draws on data from both research and clinical care settings in order to contribute to a learning healthcare system, as well as incorporating new sources of “real world” data—such as lifestyle and environmental exposure data.

Regulatory frameworks governing the MIC should provide consistent rights and protections to all participants and data contributors, regardless of the circumstances leading to their involvement in the MIC, and should accommodate both clinical and scientific uses of shared data. For example, the Health Insurance Portability and Accountability Act (HIPAA) Privacy Rule guarantees individuals a right to access and receive copies of their health record data, yet this right remains poorly understood, difficult to exercise, and only covers the portion of a person’s data held by traditional healthcare providers and other HIPAA-regulated entities [6, 7]. Accordingly, in the USA, data exclusively residing in research files (rather than health records) are not subject to HIPAA’s individual access right unless the research laboratory is part of a larger HIPAA-regulated covered entity [11]. This disparate treatment of research and clinical data may confuse participants who want to contribute their data to the MIC and does not make sense in the context of translational genomic research, where sequence data are stored in research files but have clinical implications.

### 7. Changes in technology and in the scale and scope of data sharing demand reconsideration of current policy frameworks related to privacy and security

Rich, integrated, multifactorial datasets linking people’s genomic, clinical, and environmental/lifestyle data are inherently susceptible to re-identification, even when de-identified according to current standards. This concern will only intensify as better infrastructure solutions enable disparate data and tissue resources and large datasets, in general, to become linked [12]. In keeping with a commitment to transparency, educating participants and the public about the potential for re-identification—without exaggerating the level of risk or losing sight of the benefits of data sharing—is important. Few things could destroy participants’ trust in the MIC more quickly and completely than unforeseen data breaches and re-identification. While re-identification risks cannot be eliminated, their likelihood can be reduced by: (1) requiring more complete accountings of disclosures and downstream uses of data in de-identified forms, (2) developing laws and regulations that distinguish benign uses of re-identification from nefarious ones, and (3) implementing stronger sanctions and enforcement mechanisms for data misuse [13]. Finally, there are indications that individuals differ in their degree of concern about privacy, and in how they view trade-offs between risk and benefit in the context of data sharing to facilitate research and improvements in public health [14, 15]. These differences may justify the use of data-sharing models that give participants more control over the level of risk they are willing to incur [15–18].

### 8. Distinguishing data ownership from data access and control is critical. Notions of unitary, exclusive property rights to data run counter to building the MIC

Individuals may believe they alone “own” their healthcare and related personal data; however, exclusive ownership that is typically, and often inaccurately, associated with land and physical objects is especially misplaced in the context of the MIC, where multiple copies of data exist in multiple places. The legal status of this information is not entirely clear. In the USA, courts and legislatures have rejected individuals’ exclusive ownership claims to both biospecimens [19–21] and data [6]. Nonetheless, individuals do have recognized rights and interests related to health and genomic data. For example, individuals have a right under the HIPAA Privacy Rule to access and retain copies of their health data. However, since they do not exclusively own those data, they cannot prevent care delivery institutions from retaining copies of the data as they are legally obligated to do so in order to have a proper record of each patient’s care. In addition, the concept of exclusive ownership is in direct tension with the notion of a commons and is antithetical to the goals of the MIC. Governance structures in the MIC should focus on these non-mutually exclusive rights and interests, as well as legal and moral concepts such as trusted, custodial, and fiduciary relationships.

## Conclusions

Progress in biomedical research and movement toward a learning health system that can fully take advantage of precision medicine will depend on building a robust MIC. The challenges are many and substantial. We propose eight principles that, if built into data-sharing infrastructure and practices, can improve prospects for developing a trusted MIC. While there is a moral obligation to use the data and a duty toward the people who are contributing the data, the moral imperative alone is insufficient to make data sharing successful and sustainable. There must be standard approaches to policy and governance of data initiatives in the MIC ecosystem (principle 1) that bring together data from diverse individuals (principle 2). It is essential that participants reside at the center of the MIC (principle 3), under-represented populations are engaged (principle 4), and there is investment in efforts beyond informed consent to build and sustain trust (principle 5). Finally, legal, regulatory, and technical barriers and enablers for data sharing must also be considered and updated (principles 6–8).

These eight core principles provide a framework for advancing the policy response to data-sharing opportunities and challenges. If these principles are followed, the resulting MIC can promote broader data use (for both clinical applications and the advancement of research interests), be more inclusive and result in more diverse participation, and accrue more benefits and avoid informational harms to participants. Adoption of these principles by stakeholders will increase the likelihood that the MIC ecosystem will fulfill the promise of precision medicine.

## Abbreviations

HIPAA: Health Insurance Portability and Accountability Act
MIC: Medical information commons
